# Effect of Statins on Venous Thromboembolic Events: A Meta-analysis of Published and Unpublished Evidence from Randomised Controlled Trials

**DOI:** 10.1371/journal.pmed.1001310

**Published:** 2012-09-18

**Authors:** Kazem Rahimi, Neeraj Bhala, Pieter Kamphuisen, Jonathan Emberson, Sara Biere-Rafi, Vera Krane, Michele Robertson, John Wikstrand, John McMurray

**Affiliations:** 1 George Centre for Healthcare Innovation, University of Oxford, Oxford, United Kingdom; 2 Department of Cardiovascular Medicine, University of Oxford, Oxford, United Kingdom; 3 Oxford University Hospitals NHS Trust, Department of Cardiology, Oxford, United Kingdom; 4 Clinical Trial Service Unit and Epidemiological Studies Unit, University of Oxford, Oxford, United Kingdom; 5 Department of Vascular Medicine, University Medical Center Groningen, Groningen, The Netherlands; 6 Department of Vascular Medicine, Academic Medical Center, Amsterdam, The Netherlands; 7 Department of Internal Medicine, Division of Nephrology, University of Würzburg, Würzburg, Germany; 8 Robertson Centre for Biostatistics, University of Glasgow, Glasgow, Scotland; 9 Wallenberg Laboratory for Cardiovascular Research, Sahlgrenska Academy, Gothenburg University, Gothenburg, Sweden; 10 British Heart Foundation Cardiovascular Research Centre, University of Glasgow, Glasgow, United Kingdom; Leiden University Medical Center, Netherlands

## Abstract

**Background:**

It has been suggested that statins substantially reduce the risk of venous thromboembolic events. We sought to test this hypothesis by performing a meta-analysis of both published and unpublished results from randomised trials of statins.

**Methods and Findings:**

We searched MEDLINE, EMBASE, and Cochrane CENTRAL up to March 2012 for randomised controlled trials comparing statin with no statin, or comparing high dose versus standard dose statin, with 100 or more randomised participants and at least 6 months' follow-up. Investigators were contacted for unpublished information about venous thromboembolic events during follow-up. Twenty-two trials of statin versus control (105,759 participants) and seven trials of an intensive versus a standard dose statin regimen (40,594 participants) were included. In trials of statin versus control, allocation to statin therapy did not significantly reduce the risk of venous thromboembolic events (465 [0.9%] statin versus 521 [1.0%] control, odds ratio [OR] = 0.89, 95% CI 0.78–1.01, *p* = 0.08) with no evidence of heterogeneity between effects on deep vein thrombosis (266 versus 311, OR 0.85, 95% CI 0.72–1.01) and effects on pulmonary embolism (205 versus 222, OR 0.92, 95% CI 0.76–1.12). Exclusion of the trial result that provided the motivation for our meta-analysis (JUPITER) had little impact on the findings for venous thromboembolic events (431 [0.9%] versus 461 [1.0%], OR = 0.93 [95% CI 0.82–1.07], *p* = 0.32 among the other 21 trials). There was no evidence that higher dose statin therapy reduced the risk of venous thromboembolic events compared with standard dose statin therapy (198 [1.0%] versus 202 [1.0%], OR = 0.98, 95% CI 0.80–1.20, *p* = 0.87). Risk of bias overall was small but a certain degree of effect underestimation due to random error cannot be ruled out.

*Please see later in the article for the Editors' Summary.*

**Conclusions:**

The findings from this meta-analysis do not support the previous suggestion of a large protective effect of statins (or higher dose statins) on venous thromboembolic events. However, a more moderate reduction in risk up to about one-fifth cannot be ruled out.

## Abstract 2

A systematic review and meta-analysis conducted by Kazem Rahimi and colleagues re-evaluates the hypothesis, generated in previous studies, that statins may reduce the risk of venous thromboembolic events. Their meta-analysis does not support the previous findings.

## Editors' Summary

**Background:**

Blood normally flows smoothly throughout the human body, supplying its organs and tissues with oxygen and nutrients. But, when an injury occurs, proteins called clotting factors make the blood gel (coagulate) at the injury site. The resultant blood clot (thrombus) plugs the wound and prevents blood loss. Occasionally, however, a thrombus forms inside an uninjured blood vessel and partly or completely blocks the blood flow. A clot inside one of the veins (vessels that take blood towards the heart) deep within the body is called a deep vein thrombosis (DVT). Symptoms of DVT (which usually occurs in the leg) include pain, swelling, and redness in the affected limb. DVT is treated with heparin and warfarin, two anticoagulant drugs that stop the blood clot growing. If left untreated, part of the clot (an embolus) can break off and travel to the lungs, where it can cause a pulmonary embolism (PE), a life-threatening condition characterized by chest pain, breathlessness, coughing, and dizziness. Little is known about how to prevent DVTs and PEs but risk factors for these venous thromboembolic events include having an inherited blood clotting disorder, oral contraceptive use, having surgery, and prolonged inactivity (on long-haul plane flights, for example).

**Why Was This Study Done?:**

In 2009, a secondary (add-on) analysis of data from a randomized controlled trial (RCT, a study that randomly assigns individuals to receive different treatments and compares the outcomes associated with each treatment) called the JUPITER trial reported that rosuvastatin—a cholesterol-lowering drug (statin)—halved the risk of venous thromboembolic events among apparently healthy adults. The JUPITER trial was initiated to test whether statins reduce the risk of strokes, heart attacks, and other cardiovascular diseases (conditions that involve the heart and the blood vessels) among adults with raised levels of a predictor for these diseases called C-reactive protein; statins reduce the levels of this protein as well as those of cholesterol. Because fewer than 100 of the participants in the JUPITER trial developed a DVT or PE, the reduction in the risk of a venous thromboembolic event among the participants who took rosuvastatin could have happened by chance. In this systematic review and meta-analysis of 29 RCTs of statins that collected information on many more venous thromboembolic events, the researchers test the hypothesis that statins substantially reduce the risk of such events. A systematic review uses predefined criteria to identify all the research on a given topic; a meta-analysis is a statistical approach that combines the results of several studies.

**What Did the Researchers Do and Find?:**

The researchers identified 22 RCTs (105,759 participants) that compared the effects of statins with control (dummy) tablets and seven (40,594 participants) that compared an intensive statin regimen with a standard regimen. They then obtained largely unpublished information about the venous thromboembolic events that occurred during these trials (about 1,000 DVTs and PEs) from the original investigators. In the trials of statin versus control, allocation to statin therapy did not significantly reduce the risk of venous thromboembolic events. Thus, although events occurred in 465 participants who were given statins (0.9% of the participants) and in 521 participants who were given control tablets (1% of the participants), this difference in outcomes was not statistically significant—it could have happened by chance. Exclusion of the JUPITER trial results from the meta-analysis did not alter this finding. The researchers also found no evidence that intensive statin therapy reduced the risk of venous thromboembolic events compared to standard therapy.

**What Do These Findings Mean?:**

The findings of this meta-analysis do not support the suggestion that statins, either at the standard dose or at higher doses, reduce the risk of venous thromboembolic events substantially among healthy adults. It is possible that the effect of statins has been underestimated in this meta-analysis because of missing data or because of some other source of bias. Furthermore, because the total number of events in this meta-analysis is still relatively modest, these findings do not rule out the possibility that statins may reduce the risk of venous thromboembolic events by up to about one-fifth in some or all individuals. Additional large RCTs are now needed to investigate whether statin treatment does in fact reduce the risk of venous thromboembolic events in adults and, if it does, whether all statins have a similar effect and whether statin treatment is beneficial in everyone or only in specific subgroups of people.

**Additional Information:**

Please access these Web sites via the online version of this summary at http://dx.doi.org/10.1371/journal.pmed.1001310.

- This study is further discussed in a *PLOS Medicine* Perspective by Frits Rosendaal
- The US National Heart Lung and Blood Institute provides information on deep vein thrombosis (including an animation about how DVT causes pulmonary embolisms), and information on pulmonary embolism
- The UK National Health Service Choices website has information on deep vein thrombosis, including personal stories, on pulmonary embolism, and on statins; a Behind the Headlines article describes the JUPITER trial and its implications
- The non-profit organization US National Blood Clot Alliance provides detailed information about deep vein thrombosis and pulmonary embolism for patients and professionals and includes a selection of personal stories about these conditions
- MedlinePlus has links to further information about deep vein thrombosis, pulmonary embolism, and statins (in English and Spanish)

## Introduction

Venous thromboembolic disease (i.e., pulmonary embolism and deep vein thrombosis) is a common cause of premature death and morbidity [1]–[3], yet our knowledge about how to safely prevent it is limited. During recent years, statins have emerged as one of the most effective treatments to reduce the burden of arterial cardiovascular disease worldwide [4]. Because of their remarkably good safety profile and declining costs, there has been some interest in their potential use for prevention of other conditions, such as venous thromboembolic events [5]–[7]. Venous and arterial thrombosis often co-occur [8],[9] and seem to share some common risk factors [10]. These epidemiological findings together with experimental evidence revealing novel mechanisms for the beneficial effect of statins unrelated to their low density lipoprotein (LDL) cholesterol lowering effect [11]–[13] have raised hopes that statins may also protect against venous thromboembolic events.

Until recently, clinical evidence for the effect of statins on venous thromboembolism was largely confined to non-randomised studies (with somewhat contradictory conclusions) [14],[15]. In 2009, however, secondary analyses of the JUPITER trial, in which 17,802 apparently healthy men and women were randomly allocated to receive either rosuvastatin 20 mg daily or matching placebo, provided direct randomised evidence that statin therapy might reduce the risk of venous thromboembolic events [5]. In this trial, allocation to rosuvastatin was associated with a reduction in the risk of venous thromboembolic events of 43% (95% CI 14%–63%) during a median 1.9 y follow-up [5], but this was based on relatively few patients with a venous thromboembolic event (34 versus 60), and so may partly (or even wholly) reflect the play of chance [16]. This uncertainty was reflected by calls for confirmatory evidence from other studies [17],[18]. An opportunity to obtain such evidence is provided by the routinely collected adverse event reports in existing statin trials. We therefore performed a meta-analysis of all larger scale trials of a statin versus control, and of a more intensive versus a less intensive statin regimen [19], which collected, but did not necessarily publish, information on the incidence of venous thromboembolic events during follow-up.

## Methods

### Ethics Statement

Ethics approval was not required for this work.

### Search Strategy for Identification of Relevant Studies

PRISMA checklist is provided as Text S1. Study methods have been summarised in the study protocol (Text S2) and have been published previously [20]. In brief, we searched MEDLINE (January 1966 to March 2012), EMBASE (January 1985 to 2012 week 10), and the Cochrane Central Register of Controlled Trials (The Cochrane Library Issue 2, March 2012) for articles with a subject term “hydroxymethylglutaryl-coenzyme A reductase inhibitor” or any of the following terms: “hydroxymethylglutaryl-co A reductase inhibitor,” “statin,” “fluvastatin,” “pravastatin,” “lovastatin,” “simvastatin,” “atorvastatin,” or “rosuvastatin.” The search was limited to randomised controlled trials and human studies with no language restrictions.

### Review Methods and Selection Criteria

Four reviewers, working in pairs, independently screened all titles and abstracts for randomised controlled trials with either a parallel or factorial design, with at least one comparison of a statin versus a control regimen or a more versus less intensive statin regimen, and with a total of 100 or more randomised participants followed for at least 6 mo. Reviewing process was piloted for the first 100 abstracts by all four reviewers (KR, NB, Paul McGale, and William Majoni) to assess comparability and difficulties. Then, each abstract was independently reviewed by two researchers and disagreements resolved by retrieval of the full text article and discussion with a third person. There were no restrictions placed on participant characteristics or study outcomes. We also hand-searched the reference lists of these studies to ensure that other relevant articles, such as meta-analyses of statin trials or other types of articles related to statins and venous thromboembolic events, were not missed. After removing duplicate reports, full text articles of all remaining reports were examined.

### Data Abstraction

For each trial, the following information was recorded: study or investigator's name; mean follow-up duration; year of publication of the primary findings; randomised treatments; mean LDL cholesterol at 1 y; summary information about the studied population (number of participants, mean age, number of men, and prevalence of myocardial infarction or heart failure at randomisation); and the primary outcome of the study. The number of patients with at least one reported episode of deep vein thrombosis or pulmonary embolism was recorded. In trials where information on such outcomes had not previously been published, we asked the investigators to abstract the relevant numbers from their routine records of adverse events. Non-responders were sent at least one reminder after about 3 wk and were also contacted by telephone.

### Assessment of Risk of Bias

To identify potential sources of bias in the reported events we followed the Cochrane Collaboration's risk of bias framework [21] and considered for each trial the following risk domains: (i) selection bias (random sequence generation and allocation concealment); (ii) performance bias (blinding of participants and study investigators for the outcomes of interest); (iii) detection bias (blinding of outcome assessors); (iv) attrition bias (incomplete outcome data); (v) reporting bias (selective outcome reporting). Risk of bias for each domain was categorised as low, unclear, or high. This information was used to make judgements about the overall risk of bias for each trial. We followed the Cochrane Collaboration's recommendation to make judgements on the basis of whether the ranking of the level of bias across domains could have led to any material bias on the outcomes of interests and, where applicable, what the direction of the bias would likely be [21].

### Statistical Analysis

Our primary hypothesis was to test whether statin treatment reduced the risk of venous thromboembolic events. The primary analyses were, therefore, restricted to trials of statin versus control (i.e., placebo or usual care). However, since the anti-inflammatory effect of statins (which could be a potential mechanism for any venous anti-thrombotic effects) have been suggested to be more pronounced in high-dose statin therapy [22] and since there is some non-randomised evidence to suggest a greater reduction in risk of venous thromboembolic events with higher doses of statins [19],[23], we also performed secondary analyses on the basis of the trials that had compared a more intensive versus a standard statin regimen. Odds ratios (ORs) for each trial and summary estimates of ORs across trials were estimated using Peto's one-step method (see Text S3) [24]. Estimates of heterogeneity between trials were presented together with *I* ^2^ statistics and their confidence intervals.

We performed three subgroup analyses: (i) to estimate effects separately for pulmonary embolism and deep vein thrombosis; (ii) to estimate effects separately in trials that specifically excluded patients with a known history of vascular disease compared with other trials; and (iii) to estimate effects separately according to the type of statin tested. The summary ORs for subgroups were compared using a standard chi-squared test. In a sensitivity analysis, we performed a meta-analysis of the individual trial results weighted by the absolute LDL cholesterol difference in each trial at 1 y (Text S3) [4].

Statistical analyses were done using R version 2.11.1 [25]. All statistical tests were two-sided and all analyses were done on an intention-to-treat basis.

## Results

**Figure 1:**
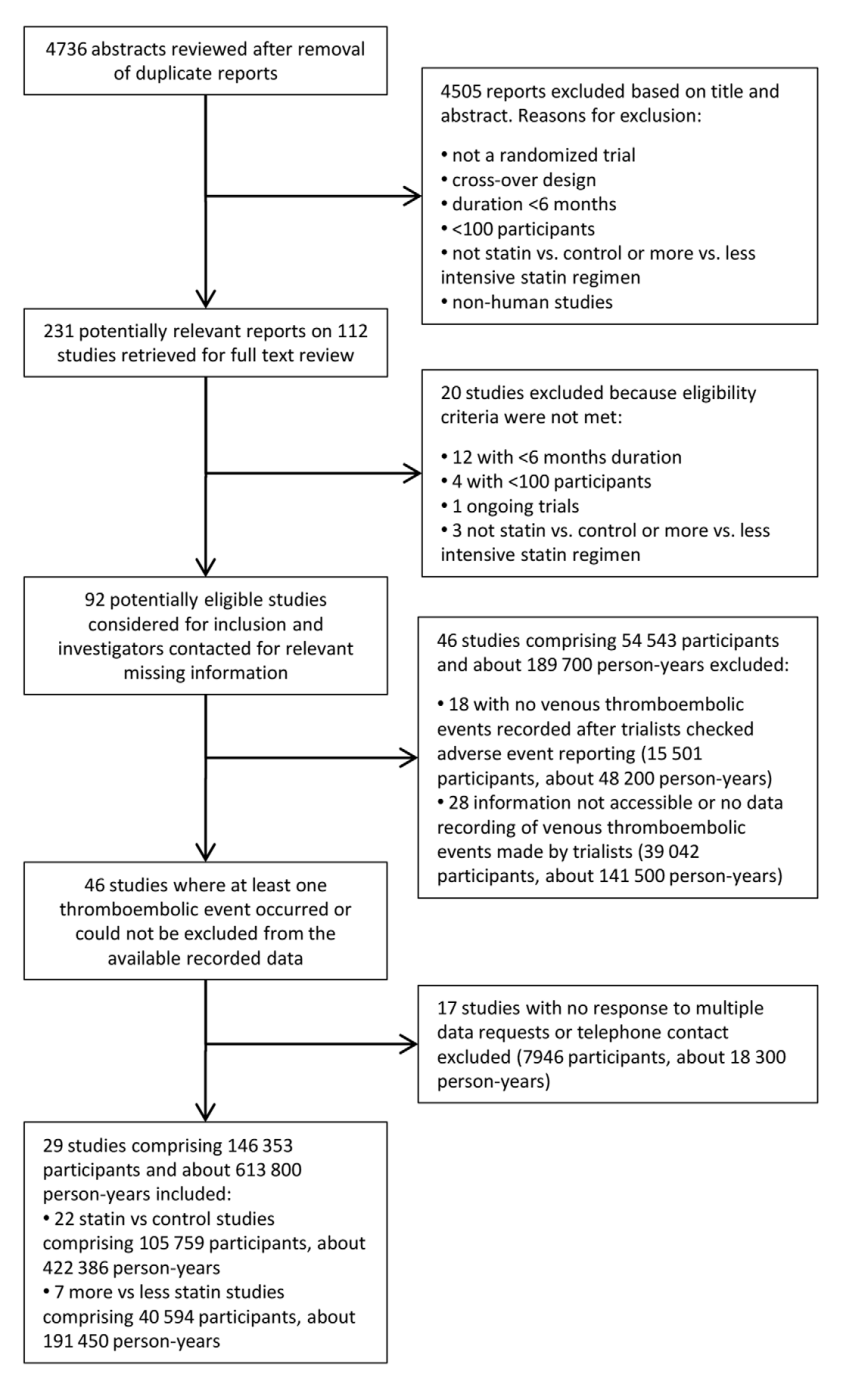
Flow-diagram of search retrieval process.

Out of 4,736 abstracts reviewed, 231 papers describing 112 trials were retrieved for further examination, of which 92 had both a follow-up duration of 6 mo or longer and had included 100 patients or more (Figure 1). Of these 92 trials, 47 trials comprising 54,643 participants and about 189,800 person-years' follow-up were excluded either because no venous thromboembolic events were recorded (i.e., zero events in both groups after interrogation of trial database) or because such information was not accessible to the trial investigators at the time. A further 16 trials comprising 7,846 participants and 18,200 person-years follow-up were excluded because there was no response to the data request. Our final database therefore included 29 trials comprising 146,353 participants and about 613,800 person-years follow-up. Of these, published information about venous thromboembolic events was available (at the time of our database search) from just two trials [5],[26], but unpublished information was provided by authors for a further 27 trials. 22 trials compared the effect of statin with control (105,759 randomised participants and 422,000 person-years follow-up) [26]–[46], and seven trials compared a more intensive with a standard dose statin (40,594 randomised participants and 191,000 person-years follow-up) [47]–[53], with no overlap between the two trial groups [39]. The characteristics of the 29 included trials are shown in Table 1. Risk of bias for individual trials is summarised in Table 2. Risk of bias for venous thromboembolic events was deemed low for all included trials.

**Table 1:** Summary of trials characteristics.

Study	Year of Publication of Main Results	Mean Follow-up (y)	Country/Region	Treatment Comparison		LDL-c Difference (mmol/l)^a^	Population Characteristics			
				Intervention	Control Regimen		Main Inclusion Criteria	Total *n* Participants	Mean Age (y)	Male (%)
**Statin versus control regimen**										
AFCAPS/TexCAPS [27]	1998	5.3	USA	L 20–40 mg	Placebo	0.94	Primary prevention	6,605	58	85
LIPID [28]	1998	5.6	Australia, New Zealand	P 40 mg	Placebo	1.03	History of MI or UA	9,014	62	83
HPS [29]	2002	5.0	UK	S 40 mg	Placebo	1.29	Vascular disease or diabetes	20,536	64	75
PROSPER [26]	2002	3.2	Scotland, Ireland, Netherlands	P 40 mg	Placebo	1.04	Elderly with vascular disease or high risk	5,699	75	47
ASCOT-LLA [30]	2003	3.2	Nordics and UK	A 10 mg	Placebo	1.07	Hypertension plus other risk factor	10,305	65	81
ALERT [31]	2003	5.1	Multinational	F 40 mg	Placebo	0.84	Renal transplant recipients	2,102	50	66
CARDS [32]	2004	3.9	UK, Ireland	A 10 mg	Placebo	1.14	Type 2 diabetes plus other risk factor	2,838	62	68
PREVEND IT [33]	2004	3.8	Netherlands	P 40 mg	Placebo	1.00	Microalbuminuric patients	864	51	65
ALLIANCE [34]	2004	4.3	USA	A 10–80 mg	Usual care	1.16	CHD	2,442	61	82
4D [35]	2005	3.9	Germany	A 20 mg	Placebo	0.89	Diabetic hemodialysis patients	1,255	66	54
SALTIRE [36]	2005	2.2	UK	A 80 mg	Placebo	1.74	Calcific aortic stenosis	155	68	70
MEGA [37]	2006	5.3	Japan	P 10–20 mg	No treatment	0.67	Primary prevention	7,832	58	30
ASPEN [38]	2006	4.3	Multinational	A 10 mg	Placebo	0.99	Type 2 diabetes	1,864	61	66
SPARCL [39]	2006	4.9	Multinational	A 80 mg	Placebo	1.43	Stroke or TIA, no CHD	4,731	63	60
CORONA [40]	2007	2.7	Multinational	R 10 mg	Placebo	1.61	Ischemic heart failure	5,011	73	76
Sola et al. [41]	2007	1.0	USA	A 20 mg	Placebo	0.81	Nonischemic heart failure	108	54	33
JUPITER [5]	2008	1.9	Multinational	R 20 mg	Placebo	1.09	Primary prevention	17,802	66	62
GISSI-HF [42]	2008	3.9	Italy	R 10 mg	Placebo	0.92	CHF	4,574	68	77
METEOR [43]	2009	2.0	Multinational	R 40 mg	Placebo	1.79	Primary prevention	981	60	57
LEADe [44]	2010	1.5	Multinational	A 80 mg	Placebo	0.30	Mild to moderate probable Alzheimer disease	640	74	48
ASTRONOMER [45]	2010	3.5	Canada	R 40 mg	Placebo	1.73	Mild to moderate aortic stenosis	269	58	61
LORD [46]	2010	2.5	Australia	A 10 mg	Placebo	0.80	Chronic kidney disease	132	62	65
**More versus less intensive statin therapy**										
ASAP [47]	2001	2.0	Netherlands	A 80 mg	S 40 mg	0.62	Familial hypercholesterolaemia	330	48	40
A-Z [48]	2004	2.0	Multinational	S 80 mg	S 20 mg	0.30	Acute coronary syndrome	4,497	61	75
REVERSAL [49]	2004	1.5	USA	A 80 mg	P 40 mg	0.97	>20% stenosis on routine coronary angiogram	657	56	72
PROVE IT [50]	2004	2.0	Multinational	A 80 mg	P 40 mg	0.65	Acute coronary syndrome	4,162	58	78
TNT [51]	2005	4.9	Multinational	A 80 mg	A 10 mg	0.62	Clinically evident CHD	10,001	61	81
IDEAL [52]	2005	4.8	Nordics, Netherlands, Iceland	A 80 mg	S 20 mg	0.55	MI	8,888	62	81
SEARCH [53]	2010	6.7	UK	S 80 mg	S 20 mg	0.39	MI	12,064	62	81

a LDL-cholesterol differences are based on average differences between the two groups at 1 y (or the closest time to 1 y if 1 y data unavailable).

A, atorvastatin; CABG, coronary artery bypass graft surgery; CHD, coronary heart disease; CHF, chronic heart failure; L, lovastatin; MI, myocardial infarction; P, pravastatin; R, rosuvastatin; S, simvastatin; TIA, transient ischaemic attack.

**Table 2:** Outcome determination and risk of bias for venous thromboembolic events.

Study	VTE Outcome Determination		Selection Bias	Performance Bias	Detection Bias	Attrition Bias	Reporting Bias	Overall Risk of Bias
	Adjudicated	Reported as Adverse Event Only						
Statin versus control regimen								
AFCAPS/TexCAPS [27]	No	Yes	Low	Low	Low	Low	Low	Low
LIPID [28]	No	Yes	Low	Low	Low	Low	Low	Low
HPS [29]	No	Yes	Low	Low	Low	Low	Low	Low
PROSPER [26]	Yes	Yes	Low	Low	Low	Low	Low	Low
ASCOT-LLA [30]	No	Yes	Low	Low	Low	Low	Low	Low
ALERT [31]	No	Yes	Low	Low	Low	Low	Low	Low
CARDS [32]	No	Yes	Low	Low	Low	Low	Low	Low
PREVEND IT [33]	No	Yes	Low	Low	Low	Low	Low	Low
ALLIANCE [34]	No	Yes	Unclear	Low	Low	Low	Low	Low
4D [35]	No	Yes	Low	Low	Low	Low	Low	Low
SALTIRE [36]	No	Yes	Unclear	Low	Low	Low	Low	Low
MEGA [37]	No	Yes	Low	Low	Low	Low	Low	Low
ASPEN [38]	No	Yes	Low	Low	Low	Low	Low	Low
SPARCL [39]	No	Yes	Low	Low	Low	Low	Low	Low
CORONA [40]	No	Yes	Low	Low	Low	Low	Low	Low
Sola et al. [41]	No	Yes	Unclear	Low	Low	Low	Low	Low
JUPITER [5]	Yes	No	Low	Low	Low	Low	Low	Low
GISSI-HF [42]	No	Yes	Low	Low	Low	Low	Low	Low
METEOR [43]	No	Yes	Low	Low	Low	Low	Low	Low
LEADe [44]	No	Yes	Low	Low	Low	Low	Low	Low
ASTRONOMER [45]	No	Yes	Low	Low	Low	Low	Low	Low
LORD [46]	No	Yes	Low	Low	Low	Low	Low	Low
More versus less intensive statin therapy								
ASAP [47]	No	Yes	Unclear	Low	Low	Low	Low	Low
A-Z [48]	No	Yes	Low	Low	Low	Low	Low	Low
REVERSAL [49]	No	Yes	Low	Low	Low	Low	Low	Low
PROVE IT [50]	No	Yes	Low	Low	Low	Low	Low	Low
TNT [51]	No	Yes	Unclear	Low	Low	Low	Low	Low
IDEAL [52]	No	Yes	Low	Low	Low	Low	Low	Low
SEARCH [53]	Yes (for PE)	No	Low	Low	Low	Low	Low	Low

Selection bias is based on random sequence generation and allocation concealment; performance bias includes blinding of participants and study investigators for the outcomes of interest; detection bias includes blinding of outcome assessors; attrition bias indicates systematic loss of participants in one arm, which could lead to missing outcome data for that trial arm over the other trial arm; and reporting bias includes the possibility of selective outcome reporting. Selection bias is a feature of the trial design. Performance and detection bias are overall low given that most data were collected without any prior knowledge of the investigators of the tested hypothesis in this study at the time of event collection. All analysis in this report are based on intention-to-treat and we further mitigated the possible effect of any attrition bias and reporting bias at individual trial level by collection of additional unpublished data.

PE, pulmonary embolism; VTE, venous thromboembolic event.

**Figure 2:**
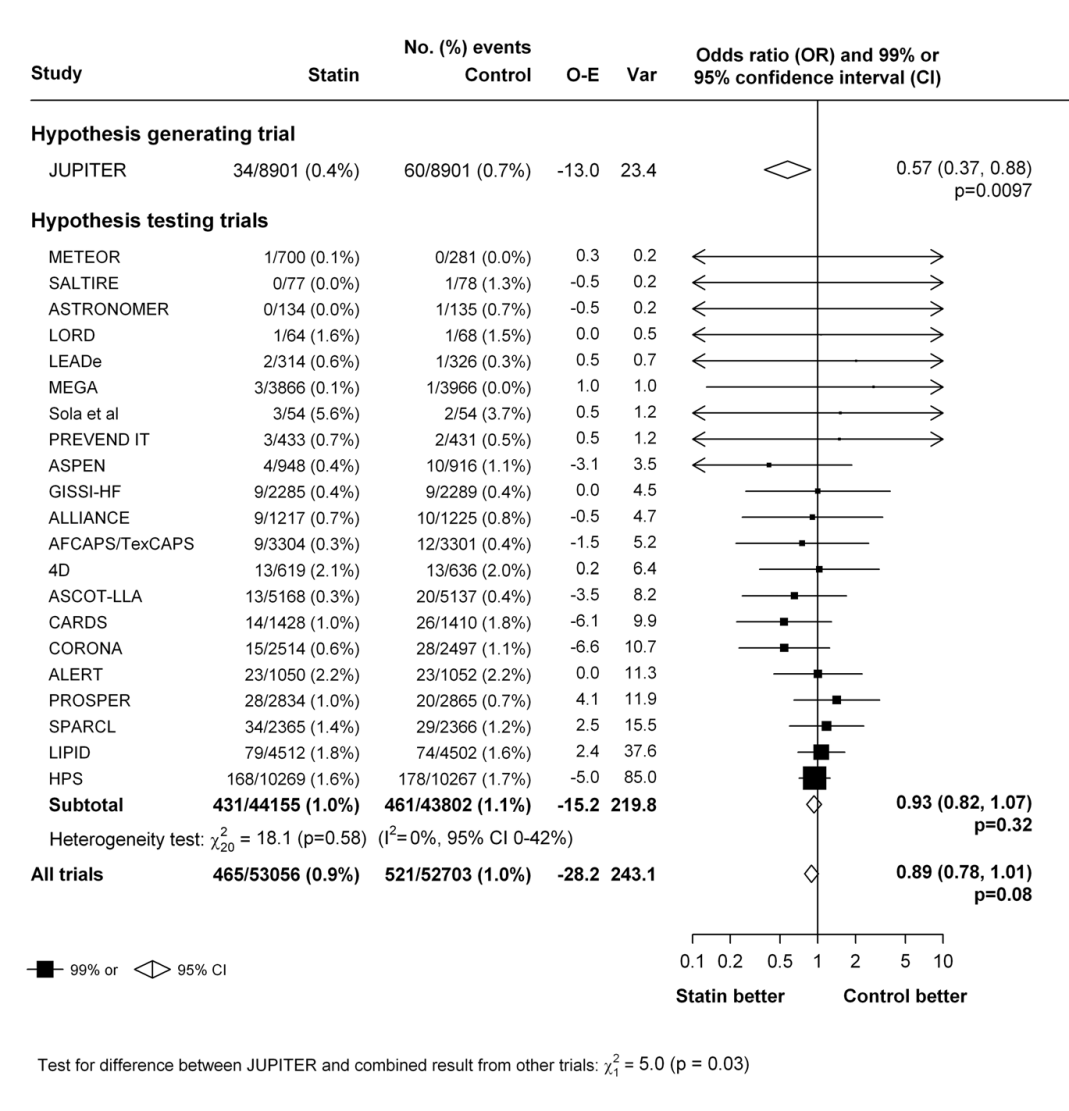
Effect of statin therapy on venous thromboembolism.

The primary analyses were restricted to the 22 trials that compared a statin with a control regimen. In these trials, an episode of venous thromboembolic event occurred in 986 patients. Overall, there was no clear evidence that statin therapy reduced the risk of venous thromboembolic events (465 [0.9%] statin versus 521 [1.0%] control, OR = 0.89 [95% CI 0.78–1.01]; *p* = 0.08) (Figure 2). There was no evidence of heterogeneity in estimated effect size between the trials (heterogeneity χ^2^_21 = _23; *p* = 0.34) but a moderate degree of statistical inconsistency between the trials could not be ruled out (*I* ^2 = ^0%, 95% CI 0%–43%). Since it was the result from the JUPITER trial that motivated us to perform this meta-analysis, it could be argued that that result should be considered only as “hypothesis generating,” and that including it in the main analyses may have led to a summary point estimate, CI, and *p*-value that are appreciably biased [54],[55]. Excluding this trial, however, had little effect on the overall result (431 [0.9%] versus 461 [1.0%], OR 0.93; 95% CI 0.82–1.07; *p* = 0.32) (Figure 2). Virtually identical results were seen when the individual trial results were weighted by the 1-y LDL cholesterol difference (in all 22 trials: OR 0.90 per mmol/l LDL cholesterol reduction, 95% CI 0.81–1.00; *p* = 0.05).

**Figure 3:**
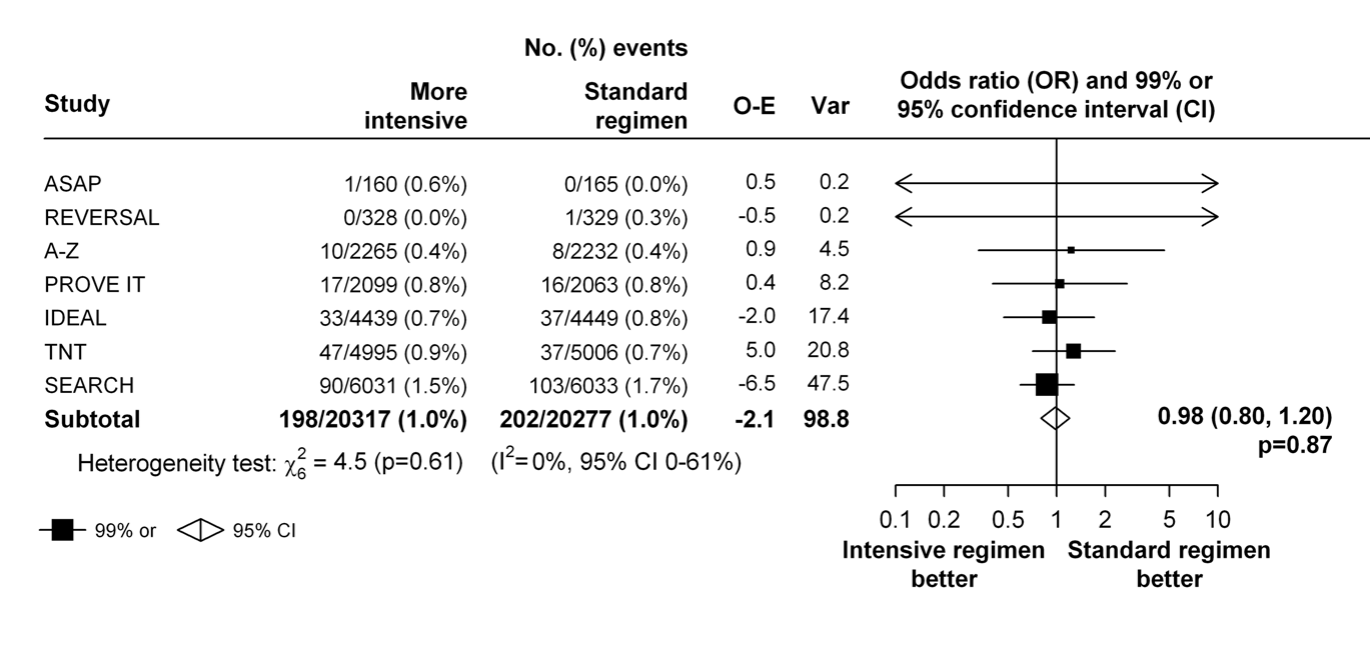
Effect of more intensive versus standard statin therapy on venous thromboembolism.

In the seven trials that compared a more intensive versus a standard statin regimen, there was no evidence that higher dose statin therapy reduced the risk of venous thromboembolic events compared with standard dose statin therapy (198 [1.0%] versus 202 [1.0%], respectively, OR 0.98; 95% CI 0.80–1.20; *p* = 0.87) and there was no evidence that the effect varied within these trials (heterogeneity χ_6_^2 = ^4.5; *p* = 0.61) (Figure 3). However a moderate to large degree of statistical inconsistency between the trials could not be ruled out (*I* ^2 = ^0%, 95% uncertainty level 0%–61%). The effect estimates weighted for 1-y LDL cholesterol differences were similar (weighted OR 0.99, 95% CI 0.66–1.51, per 1 mmol/l LDL cholesterol reduction).

**Figure 4:**
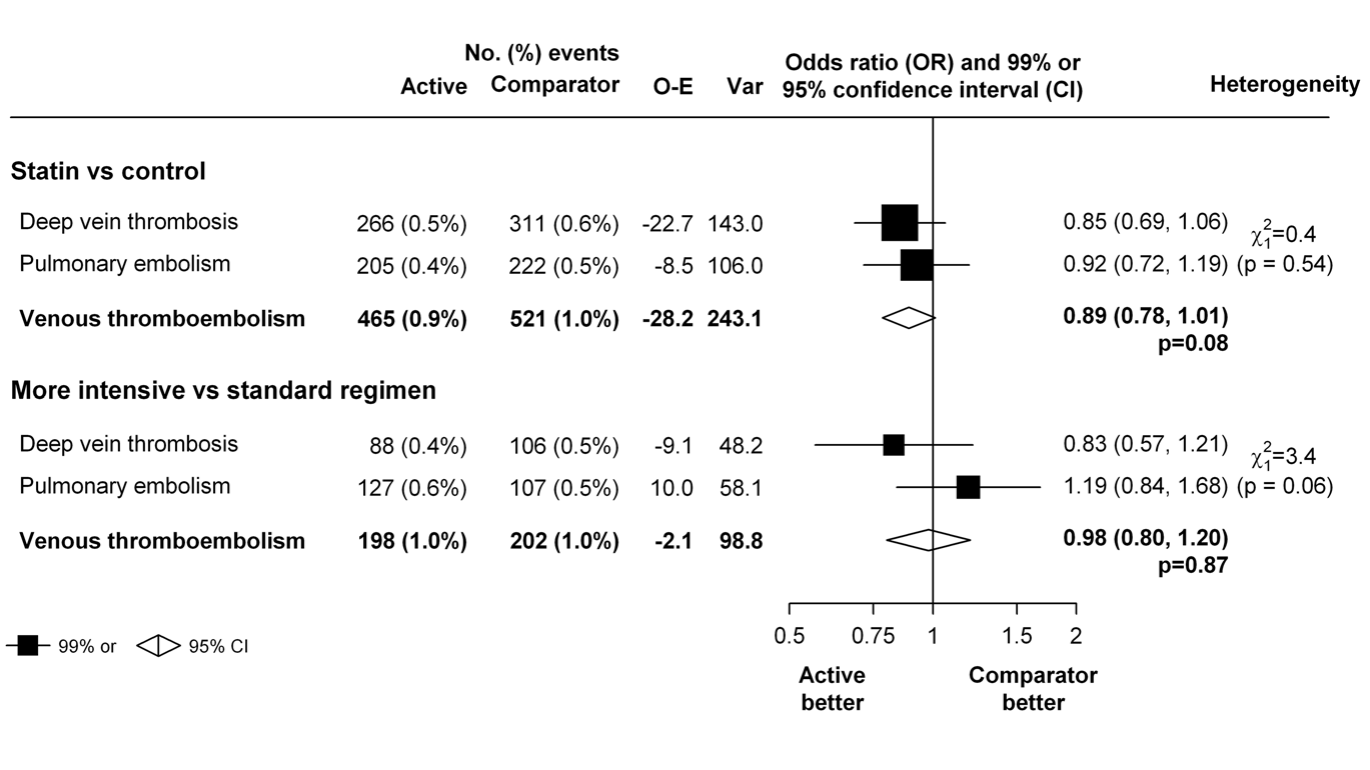
Effect of statin therapy on separate components of venous thromboembolism.

To assess a possible differential effect of statins (or higher dose statins) on pulmonary embolism and deep vein thrombosis, we estimated the effects on each outcome separately. There was no good evidence that the effect of statin therapy differed by the type of outcome (χ^2^_1 = _0.4, *p* = 0.54 for heterogeneity for the trials of statin versus control, and χ^2^_1 = _3.4, *p* = 0.06 for heterogeneity for the trials of more intensive versus standard dose statin) (Figure 4). Nor was there evidence that either statin therapy or higher dose statin therapy significantly reduced the risk of either type of outcome in isolation (deep vein thrombosis: 266 versus 311, OR 0.85 [99% CI 0.69–1.06] for trials of statin versus control and 88 versus 106, OR 0.83 [99% CI 0.57–1.21] for trials of more versus less statin; pulmonary embolism: 205 versus 222, OR 0.92 [99% CI 0.72–1.19] for trials of statin versus control and 127 versus 107, OR 1.19 [99% CI 0.84–1.68] for trials of more versus less statin) (Figure 4).

**Figure 5:**
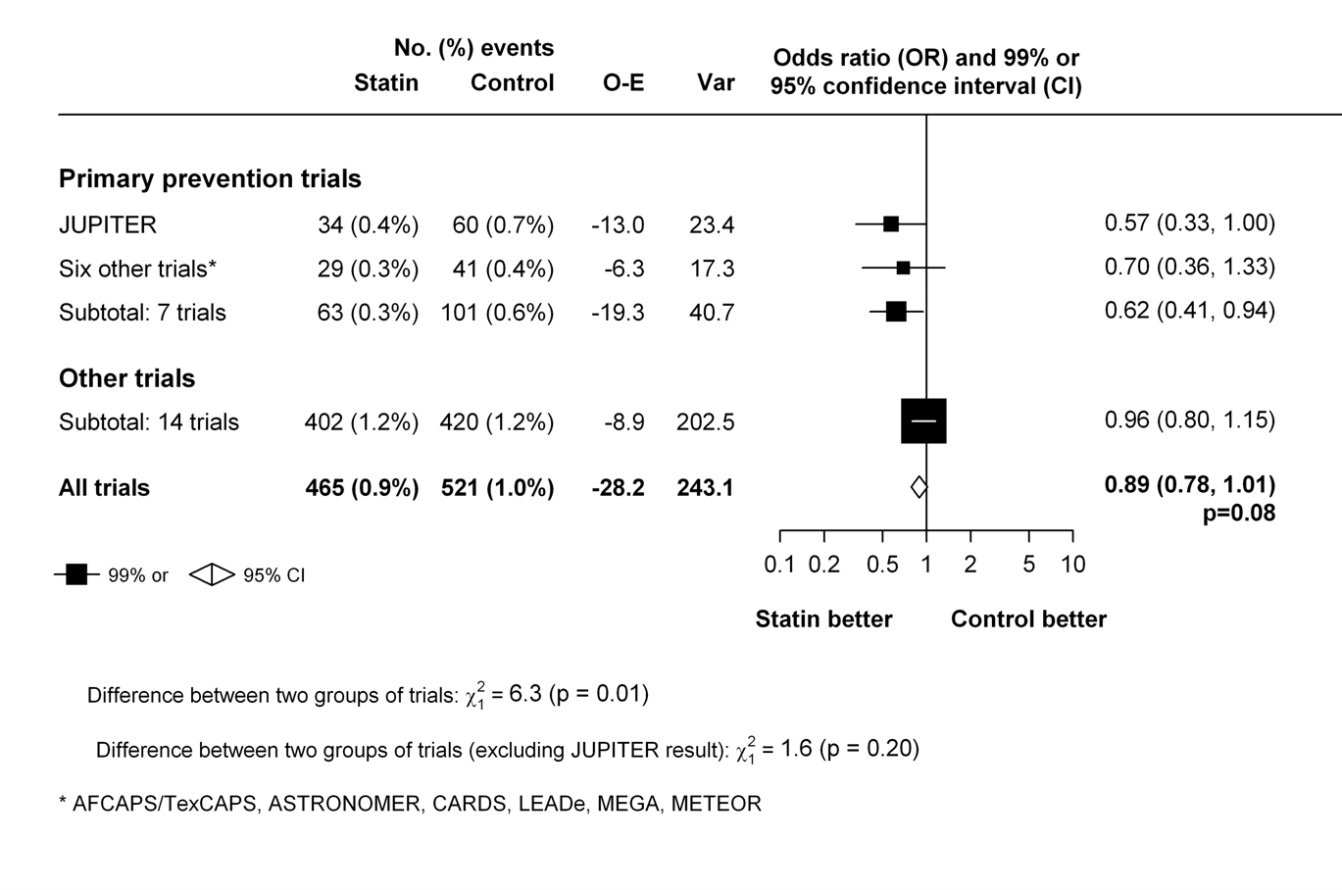
Effect of statin therapy on venous thromboembolism in primary cardiovascular prevention trials compared with other trials.

In trials that exclusively studied patients with no previous history of cardiovascular disease [5],[27],[32],[37],[43]–[45], statin therapy was associated with a significant 38% reduction in the risk of venous thromboembolism (OR 0.62, 99% CI 0.41–0.94), which appeared to differ from the non-significant 4% reduction (OR 0.96, 99% CI 0.80–1.15) seen in other trials (*p*-value for heterogeneity between two groups of trials = 0.01) (Figure 5). However, there were no significant differences between the two groups of trials when the hypothesis-generating JUPITER trial was excluded from these analyses (*p*-value for heterogeneity between two groups of trials after exclusion of JUPITER = 0.20).

There was no good evidence that the effect of statin therapy on venous thromboembolism varied depending on type of statin studied (χ^2^_5 = _10.7, *p* = 0.06) (Figure S1), particularly when the results from the JUPITER trial were excluded (χ^2^_5 = _5.8, *p* = 0.32) (Figure S2).

## Discussion

In this study, we gathered information from over 100,000 participants in 22 randomised trials of statin therapy versus control and 40,000 participants in seven randomised trials of intensive versus standard dose statin therapy, which together involve about 14 times as many venous thromboembolic events as previously reported in the JUPITER study [5]. Overall, the results from this meta-analysis do not support the suggestion that statins [5] (or higher doses of statins [19],[23]) reduce the risk of venous thromboembolic events substantially, although a more moderate reduction in risk up to about one-fifth cannot be ruled out.

Our meta-analysis has several strengths compared to previous reports. Compared with previous publications that are either based on non-randomised comparisons or have been based on one single randomised trial with limited events, our findings are based on substantially more first events from randomised trials and, importantly, include previously unpublished as well as published data. Inclusion of unpublished data helps avoid the well-documented problems caused by the preferential publication of positive findings (i.e., publication bias) [16],[56]. However, there is a perception among some that such unpublished data may be of inferior quality compared with data already in the public domain, despite the lack of evidence supporting such a view [21],[57]. In our study, reports from all trials had previously been subject to external peer review and the risk of bias in these trials for the outcomes of interest was judged to be low in our assessments (Table 2). Most of the trials had a double-blind design (i.e., trial participants and investigators were unaware of the treatment allocation) and, perhaps more importantly, venous thromboembolic events had been collected routinely as part of the safety and efficacy monitoring done in each trial (at which point there were no specific hypotheses related to venous thromboembolic events) [26]. The use of routine unadjudicated events from these properly designed and conducted randomised trials is also unlikely to have resulted in any substantial biases because any under- or over-reporting of events (or, for that matter, lack of independent confirmation) would be expected to affect both treatment arms equally [20],[58],[59]. While systematic over-reporting of events unaffected by statin therapy would tend to bias treatment effects towards the null, the magnitude of the bias would again likely be small because OR estimates are surprisingly robust to such errors. For example, even if as much as one-quarter of the reported venous thromboembolic events in our study were “false” events that were unaffected by statin treatment (and hence equally distributed by treatment group), the estimate of the OR among the trials of statin versus control would have been expected to change from 0.89 (shown in Figure 2) to 0.86 as result of removal of the false events. Confirmation of diagnosis of symptomatic venous thromboembolism in routine clinical settings already depends on objective measures such as imaging and biochemistry. It therefore seems implausible that over-reporting of unrelated outcomes in these trials of the magnitude needed to result in substantial bias in OR estimates would have occurred. This is further supported by a recent study in which treatment effects based on adjudicated versus unadjudicated vascular events in ten trials involving over 9,000 events were compared, and found to be virtually identical (OR for reported versus adjudicated outcomes 1.00, 95% CI 0.97–1.02) [59]. Indeed, if anything, one might expect the reported outcomes in our study to be more consistent with “real-world” settings in effectiveness studies and, therefore, more relevant to policy and practice.

Another concern about inclusion of unpublished data in meta-analysis is incompleteness in trials gathered, which may itself become a source of bias [57]. In our study, we sought to obtain data from all larger trials, including repeated attempts at contacting study authors. However, we might still have missed relevant event information from at least 16 further trials where there was no response from investigators (in another 47 trials, either no events occurred or events were not available to the investigators themselves so would therefore not constitute a source of bias). However, these 16 trials would have contributed only about an additional 18,000 person-years of exposure (compared to about 600,000 person-years of exposure in the 29 included trials). In addition, if an important reduction in venous thromboembolism had been observed in any single trial for which data were not made available, it seems likely that the result may already have been published (as most of these trials were completed several years ago), and hence identified by our literature search. Thus, the relatively small amount of missing information is unlikely to have resulted in any material change to our conclusions. This is illustrated by a recent similar study that assessed the effect of statin on atrial fibrillation [20]. Additional data on this outcome from a larger statin trial became available only after the meta-analysis that combined both published and unpublished data were published and this new information was entirely consistent with the pooled evidence [60].

Nonetheless, it should be recognised that the total number of first events in our meta-analysis (about 1,000 first reports of venous thromboembolism) is still relatively modest, as reflected by the confidence intervals that are consistent with anything from no effect to a real reduction in risk of about one-fifth. This makes the results of any subgroup analyses particularly unreliable, and so they need to be interpreted with due caution [54]. Consequently, this study cannot reliably investigate the potential differential effect of statins (or higher dose statins) in certain subgroups of patients based on important baseline characteristics such as prior history of venous thromboembolic events and use of anti-platelet or anti-coagulant therapy, or by the underlying cause of such events (e.g., provoked by cancer or other events versus unprovoked events).

Similarly, the suggested heterogeneity in effects on venous thromboembolism by prior vascular disease (Figure 5) is far from definitive. While it may be considered biologically plausible for there to be a proportionally smaller effect among people with pre-existing vascular disease or other chronic conditions (if the risk of venous thromboembolic events were less amenable to statin therapy as the proportion of events that are provoked by causes such cancer or immobility increases), the difference seen between trials that included healthier populations and those that did not was not statistically significant when the hypothesis-generating JUPITER trial was excluded (Figure 5). The same observation was made with regards to type of statins used (Figures S1 and S2). Thus, to demonstrate such differences (if one were to exist) would require further evidence from randomised trials.

Might the results be biased in favour of statin therapy owing to the interdependency of venous and arterial thrombosis? Statins reduce the risk of arterial vascular events (including hospitalisations) substantially [4], and more intensive regimens produce further definite reductions in risk [61]. One might therefore expect the number of venous thromboembolic events (some of which are related to arterial cardiovascular events [8],[9]) to have been greater among patients allocated placebo/less intensive statin regimens. While this may be plausible, it seems likely that the incidence of venous thromboembolic events following a cardiovascular event would be low (for example only six out of the 94 events in JUPITER occurred following a cardiovascular event) and so unlikely to have introduced any substantial bias in favour of statins.

In conclusion, this study provides a more detailed assessment of the potential effects of statins (or higher dose statins) on venous thromboembolic events than has previously been possible. We were unable to confirm the large proportional reduction in risk suggested by some previous studies. However, a more modest but perhaps clinically worthwhile reduction in venous thromboembolic events in some or all types of patient cannot be ruled out.

## Supporting Information

Text S1 PRISMA checklist.
(DOC)

Text S2 Study protocol.
(DOC)

Text S3 Statistical methods.
(DOCX)

Figure S1 Effect of statin therapy on venous thromboembolism, by type of statin.
(TIF)

Figure S2 Effect of statin therapy on venous thromboembolism, by type of statin, excluding JUPITER trial.
(TIF)

## Abbreviations

LDL: low density lipoprotein
OR: odds ratio

## References

[1] Silverstein MD, Heit JA, Mohr DN, Petterson TM, O'Fallon WM, et al (1998) Trends in the incidence of deep vein thrombosis and pulmonary embolism: a 25-year population-based study. Arch Intern Med 158: 585–593. 9521222 10.1001/archinte.158.6.585

[2] Heit JA (2005) Venous thromboembolism: disease burden, outcomes and risk factors. J Thromb Haemost 3: 1611–1617. 16102026 10.1111/j.1538-7836.2005.01415.x

[3] Naess IA, Christiansen SC, Romundstad P, Cannegieter SC, Rosendaal FR, et al (2007) Incidence and mortality of venous thrombosis: a population-based study. J Thromb Haemost 5: 692–699. 17367492 10.1111/j.1538-7836.2007.02450.x

[4] Cholesterol Treatment Trialists' Collaboration (2005) Efficacy and safety of cholesterol-lowering treatment: prospective meta-analysis of data from 90,056 participants in 14 randomised trials of statins. Lancet 366: 1267–1278. 16214597 10.1016/S0140-6736(05)67394-1

[5] Glynn RJ, Danielson E, Fonseca FAH, Genest J, Gotto AM, et al (2009) A randomized trial of rosuvastatin in the prevention of venous thromboembolism. N Engl J Med 360: 1851–1861. 19329822 10.1056/NEJMoa0900241 PMC2710995

[6] Grady D, Wenger NK, Herrington D, Khan S, Furberg C, et al (2000) Postmenopausal hormone therapy increases risk for venous thromboembolic disease: The Heart and Estrogen/progestin Replacement Study. Ann Intern Med 132: 689–696. 10787361 10.7326/0003-4819-132-9-200005020-00002

[7] Ray JG, Mamdani M, Tsuyuki RT, Anderson DR, Yeo EL, et al (2001) Use of statins and the subsequent development of deep vein thrombosis. Arch Intern Med 161: 1405–1410. 11386889 10.1001/archinte.161.11.1405

[8] Prandoni P, Bilora F, Marchiori A, Bernardi E, Petrobelli F, et al (2003) An association between atherosclerosis and venous thrombosis. N Engl J Med 348: 1435–1441. 12686699 10.1056/NEJMoa022157

[9] Srensen HT, Horvath-Puho E, Pedersen L, Baron JA, Prandoni P (2007) Venous thromboembolism and subsequent hospitalisation due to acute arterial cardiovascular events: a 20-year cohort study. Lancet 370: 1773–1779. 18037081 10.1016/S0140-6736(07)61745-0

[10] Ageno W, Becattini C, Brighton T, Selby R, Kamphuisen PW (2008) Cardiovascular risk factors and venous thromboembolism: a meta-analysis. Circulation 117: 93–102. 18086925 10.1161/CIRCULATIONAHA.107.709204

[11] Undas A, Brummel-Ziedins KE, Mann KG (2005) Statins and blood coagulation. Arterioscler Thromb Vasc Biol 25: 287–294. 15569822 10.1161/01.ATV.0000151647.14923.ec

[12] Sen-Banerjee S, Mir S, Lin Z, Hamik A, Atkins GB, et al (2005) Kruppel-like factor 2 as a novel mediator of statin effects in endothelial cells. Circulation 112: 720–726. 16043642 10.1161/CIRCULATIONAHA.104.525774

[13] Tehrani S, Mobarrez F, Antovic A, Santesson P, Lins P-E, et al (2010) Atorvastatin has antithrombotic effects in patients with type 1 diabetes and dyslipidemia. Thromb Res 126: e225–e231. 20637495 10.1016/j.thromres.2010.05.023

[14] Ramcharan AS, Van Stralen KJ, Snoep JD, Mantel-Teeuwisse AK, Rosendaal FR, et al (2009) HMG-CoA reductase inhibitors, other lipid-lowering medication, antiplatelet therapy, and the risk of venous thrombosis. J Thromb Haemost 7: 514–520. 19036068 10.1111/j.1538-7836.2008.03235.x

[15] Serensen HT, Horvath-Puho E, Sogaard KK, Christensen S, Johnsen SP, et al (2009) Arterial cardiovascular events, statins, low-dose aspirin and subsequent risk of venous thromboembolism: a population-based case-control study. J Thromb Haemost 7: 521–528. 19192118 10.1111/j.1538-7836.2009.03279.x

[16] Ioannidis JPA (2005) Contradicted and initially stronger effects in highly cited clinical research. JAMA 294: 218–228. 16014596 10.1001/jama.294.2.218

[17] Evans NS, Green D (2009) ASH evidence-based guidelines: statins in the prevention of venous thromboembolism. ASH Education Program Book 2009: 273–274. 10.1182/asheducation-2009.1.273 20008210

[18] Perez A, Bartholomew JR (2010) Interpreting The JUPITER Trial: statins can prevent VTE, but more study is needed. Cleve Clin J Med 77: 191–194. 20200169 10.3949/ccjm.77a.09077

[19] Khemasuwan D, Chae YK, Gupta S, Carpio A, Yun JH, et al (2011) Dose-related effect of statins in venous thrombosis risk reduction. Am J Med 124: 852–859. 21783169 10.1016/j.amjmed.2011.04.019

[20] Rahimi K, Emberson J, McGale P, Majoni W, Merhi M, et al (2011) Effect of statins on atrial fibrillation: collaborative meta-analysis of published and unpublished evidence from randomised controlled trials. BMJ 342: d1250. 21411487 10.1136/bmj.d1250

[21] (2011) Cochrane handbook for systematic reviews of interventions. Higgins JPT, Green S, editors. Version 5.1.0 (updated March 2011) London: The Cochrane Collaboration.

[22] Nissen SE (2005) Effect of intensive lipid lowering on progression of coronary atherosclerosis: evidence for an early benefit from the reversal of atherosclerosis with aggressive lipid lowering (REVERSAL) trial. Am J Cardiol 96: 61f–68f. 16126025 10.1016/j.amjcard.2005.07.013

[23] Doggen CJ, Lemaitre RN, Smith NL, Heckbert SR, Psaty BM (2004) HMG CoA reductase inhibitors and the risk of venous thrombosis among postmenopausal women. J Thromb Haemost 2: 700–701. 15099273 10.1111/j.1538-7836.2004.00696.x

[24] Yusuf S, Peto R, Lewis J, Collins R, Sleight P (1985) Beta blockade during and after myocardial infarction: an overview of the randomized trials. Prog Cardiovasc Dis 27: 335–371. 2858114 10.1016/s0033-0620(85)80003-7

[25] R Development Core Team (2005) R: A language and environment for statistical computing. Vienna: R Foundation for Statistical Computing.

[26] Freeman D, Robertson M, Brown EA, Rumley A, Tobias E, et al (2011) Incident venous thromboembolic events in the Prospective Study of Pravastatin in the Elderly at Risk (PROSPER). BMC Geriatrics 11: 8. 21342490 10.1186/1471-2318-11-8 PMC3053238

[27] Downs JR, Clearfield M, Weis S, Whitney E, Shapiro DR, et al (1998) Primary prevention of acute coronary events with lovastatin in men and women with average cholesterol levels: results of AFCAPS/TexCAPS. Air Force/Texas Coronary Atherosclerosis Prevention Study. JAMA 279: 1615–1622. 9613910 10.1001/jama.279.20.1615

[28] (1998) Prevention of cardiovascular events and death with pravastatin in patients with coronary heart disease and a broad range of initial cholesterol levels. The Long-Term Intervention with Pravastatin in Ischaemic Disease (LIPID) Study Group. N Engl J Med 339: 1349–1357. 9841303 10.1056/NEJM199811053391902

[29] Heart Protection Study Collaborative Group (2002) MRC/BHF Heart Protection Study of cholesterol lowering with simvastatin in 20536 high-risk individuals: a randomised placebo-controlled trial. Lancet 360: 7–22. 22115874 10.1016/S0140-6736(11)61125-2 PMC3242163

[30] Sever PS, Dahlof B, Poulter NR, Wedel H, Beevers G, et al (2003) Prevention of coronary and stroke events with atorvastatin in hypertensive patients who have average or lower-than-average cholesterol concentrations, in the Anglo-Scandinavian Cardiac Outcomes Trials-Lipid Lowering Arm (ASCOT-LLA): a multicentre randomised controlled trial. Lancet 361: 1149–1158. 12686036 10.1016/S0140-6736(03)12948-0

[31] Fellstrom B, Holdaas H, Jardine AG, Holme I, Nyberg G, et al (2004) Effect of fluvastatin on renal end points in the Assessment of Lescol in Renal Transplant (ALERT) trial. Kidney Int 66: 1549–1555. 15458450 10.1111/j.1523-1755.2004.00919.x

[32] Colhoun HM, Betteridge DJ, Durrington PN, Hitman GA, Neil HA, et al (2004) Primary prevention of cardiovascular disease with atorvastatin in type 2 diabetes in the Collaborative Atorvastatin Diabetes Study (CARDS): multicentre randomised placebo-controlled trial. Lancet 364: 685–696. 15325833 10.1016/S0140-6736(04)16895-5

[33] Asselbergs FW, Diercks GFH, Hillege HL, Van Boven AJ, Janssen WMT, et al (2004) Effects of fosinopril and pravastatin on cardiovascular events in subjects with microalbuminuria. Circulation 110: 2809–2816. 15492322 10.1161/01.CIR.0000146378.65439.7A

[34] Koren MJ, Hunninghake DB (2004) Clinical outcomes in managed-care patients with coronary heart disease treated aggressively in lipid-lowering disease management clinics: The ALLIANCE study. J Am Coll Cardiol 44: 1772–1779. 15519006 10.1016/j.jacc.2004.07.053

[35] Wanner C, Krane V, Marz W, Olschewski M, Mann JF, et al (2005) Atorvastatin in patients with type 2 diabetes mellitus undergoing hemodialysis. N Engl J Med 353: 238–248. 16034009 10.1056/NEJMoa043545

[36] Cowell SJ, Newby DE, Prescott RJ, Bloomfield P, Reid J, et al (2005) A randomized trial of intensive lipid-lowering therapy in calcific aortic stenosis. N Engl J Med 352: 2389–2397. 15944423 10.1056/NEJMoa043876

[37] Nakamura H, Arakawa K, Itakura H, Kitabatake A, Goto Y, et al (2006) Primary prevention of cardiovascular disease with pravastatin in Japan (MEGA Study): a prospective randomised controlled trial. Lancet 368: 1155–1163. 17011942 10.1016/S0140-6736(06)69472-5

[38] Knopp RH, D'Emden M, Smilde JG, Pocock SJ (2006) Efficacy and safety of atorvastatin in the prevention of cardiovascular end points in subjects with type 2 diabetes: the Atorvastatin Study for Prevention of Coronary Heart Disease Endpoints in Non-Insulin-Dependent Diabetes Mellitus (ASPEN). Diabetes Care 29: 1478–1485. 16801565 10.2337/dc05-2415

[39] Amarenco P, Bogousslavsky J, Callahan A 3rd, Goldstein LB, Hennerici M, et al (2006) High-dose atorvastatin after stroke or transient ischemic attack. N Engl J Med 355: 549–559. 16899775 10.1056/NEJMoa061894

[40] Kjekshus J, Apetrei E, Barrios V, Bohm M, Cleland JG, et al (2007) Rosuvastatin in older patients with systolic heart failure. N Engl J Med 357: 2248–2261. 17984166 10.1056/NEJMoa0706201

[41] Sola S, Mir MQS, Lerakis S, Tandon N, Khan BV (2006) Atorvastatin improves left ventricular systolic function and serum markers of inflammation in nonischemic heart failure. J Am Coll Cardiol 47: 332–337. 16412856 10.1016/j.jacc.2005.06.088

[42] (2008) GISSI-HF investigators. Effect of rosuvastatin in patients with chronic heart failure (the GISSI-HF trial): a randomised, double-blind, placebo-controlled trial. Lancet 372: 1231–1239. 18757089 10.1016/S0140-6736(08)61240-4

[43] Crouse JR 3rd, Raichlen JS, Riley WA, Evans GW, Palmer MK, et al (2007) Effect of rosuvastatin on progression of carotid intima-media thickness in low-risk individuals with subclinical atherosclerosis: the METEOR Trial. JAMA 297: 1344–1353. 17384434 10.1001/jama.297.12.1344

[44] Feldman HH, Doody RS, Kivipelto M, Sparks DL, Waters DD, et al (2010) Randomized controlled trial of atorvastatin in mild to moderate Alzheimer disease. Neurology 74: 956–964. 20200346 10.1212/WNL.0b013e3181d6476a

[45] Chan KL, Teo K, Dumesnil JG, Ni A, Tam J, et al (2010) Effect of Lipid Lowering With Rosuvastatin on Progression of Aortic Stenosis: Results of the Aortic Stenosis Progression Observation: Measuring Effects of Rosuvastatin (ASTRONOMER) Trial. Circulation 121: 306–314. 20048204 10.1161/CIRCULATIONAHA.109.900027

[46] Fassett RG, Robertson IK, Ball MJ, Geraghty DP, Coombes JS (2010) Effect of atorvastatin on kidney function in chronic kidney disease: a randomised double-blind placebo-controlled trial. Atherosclerosis 213: 218–224. 20810109 10.1016/j.atherosclerosis.2010.07.053

[47] Smilde TJ, van Wissen S, Wollersheim H, Trip MD, Kastelein JJ, et al (2001) Effect of aggressive versus conventional lipid lowering on atherosclerosis progression in familial hypercholesterolaemia (ASAP): a prospective, randomised, double-blind trial. Lancet 357: 577–581. 11558482 10.1016/s0140-6736(00)04053-8

[48] De Lemos JA, Blazing MA, Wiviott SD, Lewis EF, Fox KAA, et al (2004) Early intensive vs a delayed conservative simvastatin strategy in patients with acute coronary syndromes: Phase Z of the A to Z trial. JAMA 292: 1307–1316. 15337732 10.1001/jama.292.11.1307

[49] Nissen SE, Tuzcu EM, Schoenhagen P, Brown BG, Ganz P, et al (2004) Effect of Intensive Compared with Moderate Lipid-Lowering Therapy on Progression of Coronary Atherosclerosis: A Randomized Controlled Trial. JAMA 291: 1071–1080. 14996776 10.1001/jama.291.9.1071

[50] Cannon CP, Braunwald E, McCabe CH, Rader DJ, Rouleau JL, et al (2004) Intensive versus moderate lipid lowering with statins after acute coronary syndromes. N Engl J Med 350: 1495–1504. 15007110 10.1056/NEJMoa040583

[51] LaRosa JC, Grundy SM, Waters DD, Shear C, Barter P, et al (2005) Intensive lipid lowering with atorvastatin in patients with stable coronary disease. N Engl J Med 352: 1425–1435. 15755765 10.1056/NEJMoa050461

[52] Pedersen TR, Faergeman O, Kastelein JJP, Olsson AG, Tikkanen MJ, et al (2005) High-dose atorvastatin vs usual-dose simvastatin for secondary prevention after myocardial infarction. JAMA 294: 2437–2445. 16287954 10.1001/jama.294.19.2437

[53] (2010) SEARCH Collaborative Group. Intensive lowering of LDL cholesterol with 80 mg versus 20 mg simvastatin daily in 12 064 survivors of myocardial infarction: a double-blind randomised trial. Lancet 376: 1658–1669. 21067805 10.1016/S0140-6736(10)60310-8 PMC2988223

[54] Collins R, MacMahon S (2001) Reliable assessment of the effects of treatment on mortality and major morbidity, I: clinical trials. Lancet 357: 373–380. 11211013 10.1016/S0140-6736(00)03651-5

[55] Collins R, Armitage J (2002) High-risk elderly patients PROSPER from cholesterol-lowering therapy. Lancet 360: 1618–1619. 12457780 10.1016/S0140-6736(02)11650-3

[56] Baigent C, Peto R, Gray R, Collins R (2010) Large-scale randomized evidence: trials and meta-analyses of trials. Warrell DA, Cox TM, Firth JD, editors. Oxford textbook of medicine. 5th edition. Oxford: Oxford University Press.

[57] Cook DJ, Guyatt GH, Ryan G, Clifton J, Buckingham L, et al (1993) Should unpublished data be included in meta-analyses? current convictions and controversies. JAMA 269: 2749–2753. 8492400

[58] Granger CB, Vogel V, Cummings SR, Held P, Fiedorek F, et al (2008) Do we need to adjudicate major clinical events? Clin Trials 5: 56–60. 18283081 10.1177/1740774507087972

[59] Pogue J, Walter SD, Yusuf S (2009) Evaluating the benefit of event adjudication of cardiovascular outcomes in large simple RCTs. Clin Trials 6: 239–251. 19528133 10.1177/1740774509105223

[60] Schwartz GG, Chaitman BR, Goldberger JJ, Messig M (2010) High-dose atorvastatin and risk of atrial fibrillation in patients with prior stroke or transient ischemic attack: Analysis of the Stroke Prevention by Aggressive Reduction in Cholesterol Levels (SPARCL) trial. Am Heart J 161: 993–999. 10.1016/j.ahj.2011.02.002 21570534

[61] Cholesterol Treatment Trialists' Collaboration (2010) Efficacy and safety of more intensive lowering of LDL cholesterol: a meta-analysis of data from 170,000 participants in 26 randomised trials. Lancet 376: 1670–1681. 21067804 10.1016/S0140-6736(10)61350-5 PMC2988224

