# Supplementary material for: Effect of Statins on Venous Thromboembolic Events: A Meta-analysis of Published and Unpublished Evidence from Randomised Controlled Trials
Source: PLoS Med. 2012 Sep 18;9(9):e1001310. doi: 10.1371/journal.pmed.1001310 (PMC3445446; doi:10.1371/journal.pmed.1001310)
Supplement: Text S2 — Study protocol. (DOC) [file pmed.1001310.s002.doc]

**Tabular meta-analysis of statin trials looking at the effect of such treatment on the risk of venous thromboembolic events:**

**Study protocol**

**Background**

There is compelling evidence that statins reduce the risk of atherosclerotic cardiovascular disease in a wide range of people.^1^ However, there is some controversy regarding the extent of shared pathways between arterial and venous thrombosis and whether treatments of known efficacy for one disease process have consistent benefits for the other.

In a recently published substudy of the JUPITER trial, treatment with rosuvastatin significantly reduced the occurrence of symptomatic venous thromboembolic events (VTEs) versus placebo.^2^ However, this finding was based on relatively small number of events (34 vs 60) and, hence, is prone to random error. Although published information from other large-scale randomised trials of statins on VTEs are limited, many such trials are likely to have collected such information; eg, as a part of adverse event reporting. Pooling of such previously unpublished information would offer a unique opportunity for testing the hypothesis generated by the JUPITER trial.

**Objectives**

- A tabular meta-analysis of statin trials looking at the effect of statins on VTEs
- Comparison of the pooled effect size to test this hypothesis (against the results from JUPITER as the hypothesis-generating study)
- Depending on data availability, analysis will be separated for deep venous thrombosis and pulmonary embolism
- Comparison of any effects observed with the effect on arterial cardiovascular events
- In case of any significant effect of statins on VTEs, the role of level of LDL-reduction in individual trials could be assessed (as a potential source of heterogeneity)

**Methods**

**Study eligibility:**

- Human studies including 100 or more participants with a follow-up duration of 6 months or more.
- Randomized controlled trials with parallel design
- Intervention: statins
- Control: statin of a different potency or no such treatment
- Outcomes: VTE reported or available from the investigators
- No language restrictions

**Search strategy:**

We will search MEDLINE (January 1966 to April 2009), EMBASE (January 1985 to April 2009) and the Cochrane Central Register of Controlled Trials (The Cochrane Library Issue 1, 2009) for articles with a subject term “hydroxymethylglutaryl-coenzyme A reductase inhibitor” or any of the following terms: “hydroxymethylglutaryl-co A reductase inhibitor”, “statin”, “fluvastatin”, “pravastatin”, “lovastatin”, “simvastatin”, “atorvastatin” or “rosuvastatin”. The search will be limited to randomised controlled trials with no language restrictions.

The detailed search strategy for MEDLINE is:

"Hydroxymethylglutaryl coa reductase inhibitors"[Mesh] OR ("hydroxymethylglutaryl-coa reductase inhibitors"[MeSH Terms] OR ("hydroxymethylglutaryl-coa"[All Fields] AND "reductase"[All Fields] AND "inhibitors"[All Fields]) OR "hydroxymethylglutaryl-coa reductase inhibitors"[All Fields] OR ("hydroxymethylglutaryl"[All Fields] AND "coa"[All Fields] AND "reductase"[All Fields] AND "inhibitor"[All Fields]) OR "hydroxymethylglutaryl coa reductase inhibitor"[All Fields] OR "hydroxymethylglutaryl-coa reductase inhibitors"[Pharmacological Action]) OR ("hydroxymethylglutaryl-coa reductase inhibitors"[MeSH Terms] OR ("hydroxymethylglutaryl-coa"[All Fields] AND "reductase"[All Fields] AND "inhibitors"[All Fields]) OR "hydroxymethylglutaryl-coa reductase inhibitors"[All Fields] OR "statin"[All Fields] OR "hydroxymethylglutaryl-coa reductase inhibitors"[Pharmacological Action]) OR ("fluvastatin"[Supplementary Concept] OR "fluvastatin"[All Fields]) OR ("pravastatin"[MeSH Terms] OR "pravastatin"[All Fields]) OR ("lovastatin"[MeSH Terms] OR "lovastatin"[All Fields]) OR ("simvastatin"[MeSH Terms] OR "simvastatin"[All Fields]) OR ("atorvastatin"[Supplementary Concept] OR "atorvastatin"[All Fields]) OR ("rosuvastatin"[Supplementary Concept] OR "rosuvastatin"[All Fields]) OR (("3-hydroxy-3-methylglutaryl-coenzyme A"[Supplementary Concept] OR "3-hydroxy-3-methylglutaryl-coenzyme A"[All Fields] OR "hmg coa"[All Fields]) AND inhibitor[All Fields]) AND ("humans"[MeSH Terms] AND Randomized Controlled Trial[ptyp])

Similar search for Embase and Cochrane will be developed.

**Review methods:**

In the first stage of review all titles and abstracts of the citations to be reviewed by 2 reviewers and to be classified as:

- relevant: all the following criteria are fulfilled: human RCT, more than 200 participants, statin vs control or low-dose vs high-dose statin, follow-up duration >6 months (NB: subgroup analysis for RCTs to be included since some may contain relevant information not published in the main paper)
- possibly relevant: RCTs which may fulfil the criteria for the relevant category but not all required information is included in the abstract (e.g. duration of treatment not mentioned in the abstract)
- interesting: studies that may lead to relevant articles (e.g. meta-analysis of statin trials), published papers, editorials or comments related to statins and thrombembolic events or any other studies that are interesting from methodological point of view
- not relevant

Studies that have been classified as relevant or possibly relevant will be subject to fulltext review. Different reports of the same trial will be grouped together. Possibly relevant trials will be assessed for their potential eligibility. If they do not fulfil the eligibility criteria, they will be excluded and the reason for this will be recorded. The remaining studies will be assessed for the availability of outcomes of interest. For reports that are available as PDF-files, this will be done by searching for the following terms: VTE, PE, DVT, embol, thromb, venous, and pulmon.

**Data extraction**

An electronic data abstraction form will be used to capture the following information: study name or investigator’s name; recruitment period; mean follow-up duration; year of publication of the primary findings; randomised treatment comparisons; summary information about the studied population (number of participants, mean age, number of men, and prevalence of myocardial infarction or heart failure at randomisation); the primary outcome of the study; the mean LDL-cholesterol level at randomisation and at one year follow-up (or end of the study if follow-up duration was less than a year) by treatment allocation; and the number of patients with a report of VTE (including deep venous thrombosis, fatal and non-fatal pulmonary embolus) during the trial by treatment allocation.

If studies have reported information about VTEs, such information will be extracted immediately. In order to avoid abstracting and requesting data which has been already collected as part of the arrhythmia meta-analysis, all included studies will be checked against the arrhythmia database before any new unpublished information is sought from the investigators. The data request form will be individually amended to incorporate any previously available data. In trials where information about VTE have not previously been reported, investigators will be contacted and further information on previously unpublished events with the method of capturing such events and the definition used will be requested. Non-responders will be sent a reminder after about three weeks.

**Statistical analysis**

To test the primary hypothesis that statins might reduce the risk of VTE, the main analyses will be restricted to the trials of statin versus no or a control regimen (i.e., placebo or usual care). Secondary analyses further included the trials that compared one statin with another.

For every trial, the “observed minus expected” statistic (o – e) and its variance (v) will be calculated from the number of patients that developed VTE and the total number of patients in each treatment group. These (o – e) values, one from every trial, will be summed to produce a grand total (G), with variance (V) equal to the sum of their separate variances. The value exp(G/V) is Peto’s “one-step” estimate of the odds ratio and its 95% confidence interval is given by exp(G/V ± 1.96/V). Heterogeneity between the different hypothesis-testing trials will be assessed by calculating S – G2/V, where S is the sum of [o – e]2/v for each trial, and testing this statistic against a chi square distribution with degrees of freedom equal to one less than the number of trials. Exactly the same methods were applied to the JUPITER study. The summary odds ratios from the hypothesis-generating and the hypothesis-testing studies will be compared using a standard chi square test (on 1 degree of freedom). For the statin versus control trials and, separately, for the trials of a more versus less intensive statin regimen, the average one-year LDL-cholesterol difference between the randomised groups will be calculated as a weighted average of the individual one-year differences in each trial (d), with weights equal to the variances (v) of the individual (o –e) statistics (i.e. the average difference was calculated by the sum of the values (d x v) divided by V).

Additional analyses will explore whether there was any evidence of different effect estimates being observed among trials that studied mainly people with no prior coronary heart disease, trials that studied mainly people with prior coronary heart disease but not heart failure or advanced chronic kidney disease, or trials that studied mainly people with heart failure or advanced chronic kidney disease.

**Publication policy**

Any work carried out by the trialists will be acknowledged and any publications will be circulated prior to submission for their comments.

REFERENCES

**1.** Baigent C, Keech A, Kearney PM, Blackwell L, Buck G, Pollicino C, Kirby A, Sourjina T, Peto R, Collins R, Simes R. Efficacy and safety of cholesterol-lowering treatment: prospective meta-analysis of data from 90,056 participants in 14 randomised trials of statins. *Lancet.* 2005;366(9493):1267-1278.

**2.** Glynn RJ, Danielson E, Fonseca FAH, Genest J, Gotto AM, Jr., Kastelein JJP, Koenig W, Libby P, Lorenzatti AJ, MacFadyen JG, Nordestgaard BG, Shepherd J, Willerson JT, Ridker PM. A Randomized Trial of Rosuvastatin in the Prevention of Venous Thromboembolism. *N Engl J Med.* 2009:NEJMoa0900241.
