# Supplementary material for: Effect of Statins on Venous Thromboembolic Events: A Meta-analysis of Published and Unpublished Evidence from Randomised Controlled Trials
Source: PLoS Med. 2012 Sep 18;9(9):e1001310. doi: 10.1371/journal.pmed.1001310 (PMC3445446; doi:10.1371/journal.pmed.1001310)
Supplement: Text S3 — Statistical methods. (DOCX) [file pmed.1001310.s003.docx]

**Effect of Statins on Venous Thromboembolic Events: A Meta-analysis of Published and Unpublished Evidence from Randomised Controlled Trials**

Text S3: Statistical methods:

For every trial, the “observed minus expected” statistic (o – e) and its variance (v) were calculated from the number of patients that developed venous thromboembolic events and the total number of patients in each treatment group, using standard formulae for 2x2 contingency tables. These (o – e) values, one from every trial, were summed to produce a grand total (G), with variance (V) equal to the sum of their separate variances. The value exp(G/V) is Peto’s “one-step” estimate of the odds ratio and its continuity-corrected 95% confidence interval is given by exp(G/V ± [0.5/V + 1.96/√V]).[^1^](#_ENREF_24) Odds ratios are given with 95% confidence intervals for the overall results and with 99% confidence intervals (replacing 1.96 in the formula above by 2.576) for individual trial results and subgroup results. The heterogeneity between the different trials was assessed by calculating S – G^2^/V, where S is the sum of (o – e)^2^/v for each trial, and testing this statistic against a chi-squared distribution with degrees of freedom equal to one less than the number of trials. In forest plots, trials are shown in order of the amount of statistical information they contribute to the overall result.

In a sensitivity analysis, we performed a meta-analysis of the individual trial results weighted by the absolute LDL cholesterol difference in each trial at 1 year (done by replacing o-e with d(o-e) and v with d^2^v in the formulae above, where d is the average achieved LDL cholesterol difference at one-year in each trial)

References:

1. Yusuf S, Peto R, Lewis J, Collins R, Sleight P. Beta blockade during and after myocardial infarction: An overview of the randomized trials. *Prog Cardiovasc Dis*. 1985;27:335-371
