# Supplementary figures and images for: Effect of Statins on Venous Thromboembolic Events: A Meta-analysis of Published and Unpublished Evidence from Randomised Controlled Trials
Source: PLoS Med. 2012 Sep 18;9(9):e1001310. doi: 10.1371/journal.pmed.1001310 (PMC3445446; doi:10.1371/journal.pmed.1001310)

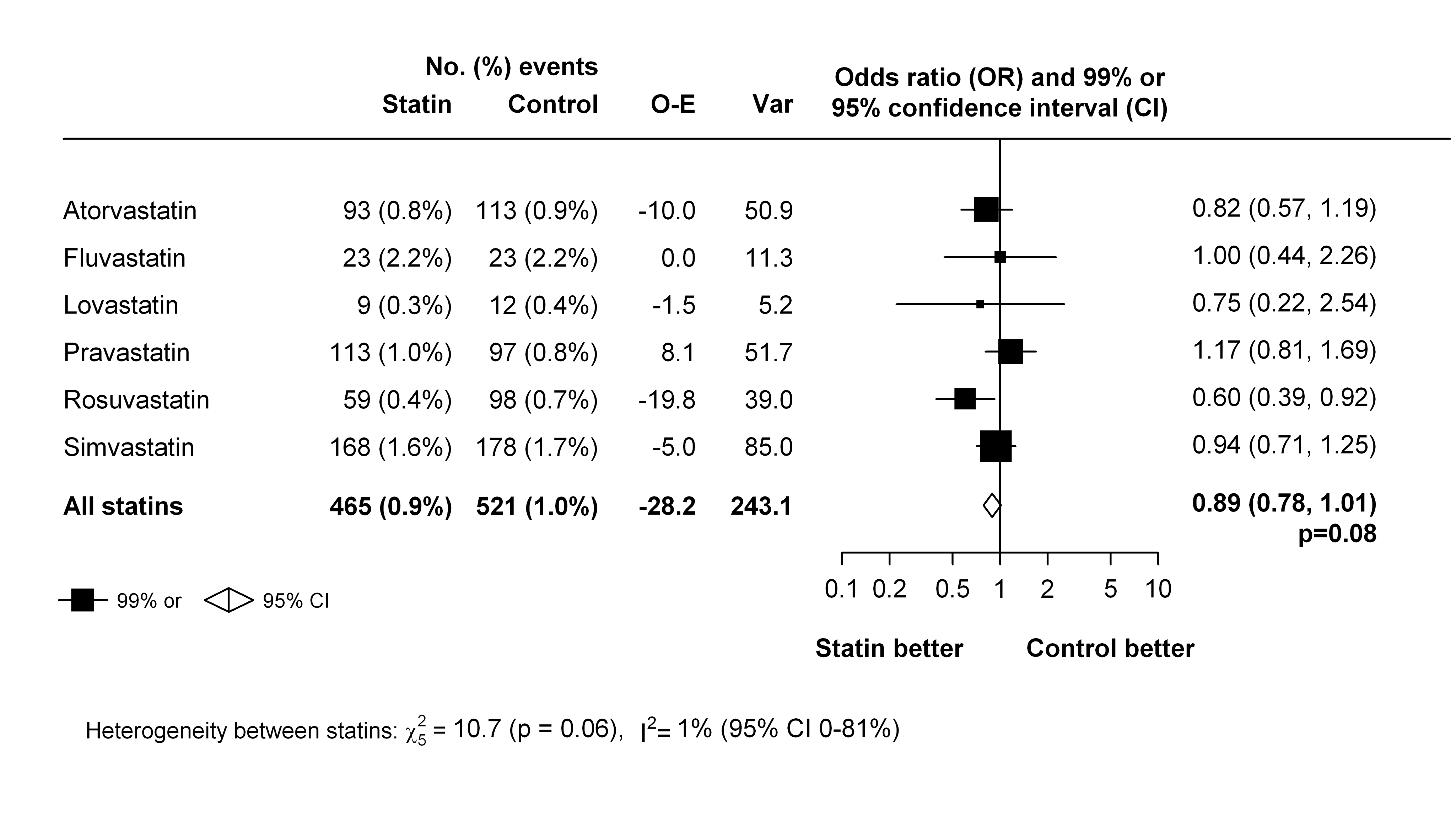

Supplement: Figure S1 — Effect of statin therapy on venous thromboembolism, by type of statin. (TIF) [file pmed.1001310.s004.tif]

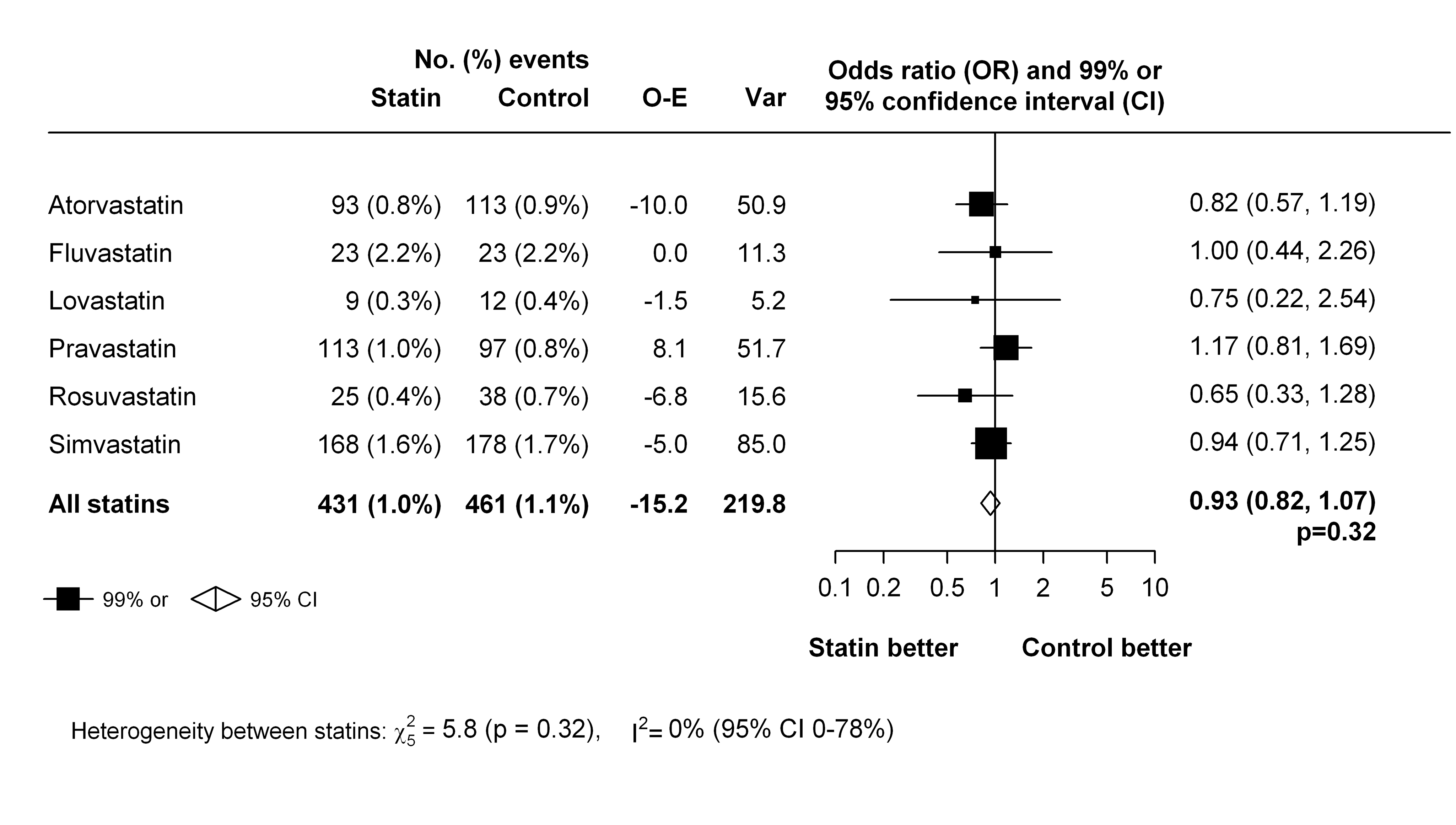

Supplement: Figure S2 — Effect of statin therapy on venous thromboembolism, by type of statin, excluding JUPITER trial. (TIF) [file pmed.1001310.s005.tif]
